# Racial Inequities in Self-Rated Health Across Brazilian Cities: Does Residential Segregation Play a Role?

**DOI:** 10.1093/aje/kwac001

**Published:** 2022-03-04

**Authors:** Joanna M N Guimarães, Goro Yamada, Sharrelle Barber, Waleska Teixeira Caiaffa, Amélia Augusta de Lima Friche, Mariana Carvalho de Menezes, Gervasio Santos, Isabel Santos, Leticia de Oliveira Cardoso, Ana V Diez Roux

**Keywords:** Brazil, interaction analysis, racial health inequities, residential segregation, self-rated health

## Abstract

Racial health inequities may be partially explained by area-level factors such as residential segregation. In this cross-sectional study, using a large, multiracial, representative sample of Brazilian adults (*n* = 37,009 individuals in the 27 state capitals; National Health Survey (*Pesquisa Nacional de Saúde*), 2013), we investigated 1) whether individual-level self-rated health (SRH) (fair or poor vs. good or better) varies by race (self-declared White, Brown, or Black) and 2) whether city-level economic or racial residential segregation (using dissimilarity index values in tertiles: low, medium, and high) interacts with race, increasing racial inequities in SRH. Prevalence of fair or poor SRH was 31.5% (Black, Brown, and White people: 36.4%, 34.0%, and 27.3%, respectively). Marginal standardization based on multilevel logistic regression models, adjusted for age, gender, and education, showed that Black and Brown people had, respectively, 20% and 10% higher prevalence of fair or poor SRH than did White people. Furthermore, residential segregation interacted with race such that the more segregated a city, the greater the racial gap among Black, Brown, and White people in fair or poor SRH for both income and race segregation. Policies to reduce racial inequities may need to address residential segregation and its consequences for health.

## Abbreviations

CI: confidence interval
PD: prevalence difference
PNS: *Pesquisa Nacional de Saúde* (National Health Survey)
PR: prevalence ratio
SES: socioeconomic status
SRH: self-rated health

Brazil is a multiracial country with the world’s fifth largest population (1). Although inequities unfavorable to people who identify their race or skin color as Black or Brown (or “Pardo”), compared with White people (1, 2)**,** have been found reported, few studies have been conducted to investigate racial inequities in health in Brazil (3). The limited focus on racial health inequities may be partially explained by the myth of a “Brazilian racial democracy,” proposed by Freyre (4) (i.e., the belief in racial egalitarianism) (3, 5, 6). The Brazilian racial democracy myth derives from the idea that in Brazil, the intermixture of Native, African, and European descendants gave rise to an interracial society in which racism is claimed to be nonexistent (5, 7).

Despite the notion of Brazilian racial democracy, there are important differences in living conditions between Black and Brown people and White people in Brazil. Historically, Black and Brown Brazilians have lower socioeconomic status (SES) and poorer health outcomes than White Brazilians (1, 8). However, individual-level differences in SES do not fully account for racial inequities in health, and in a growing body of literature from high-income countries, authors have focused on area-level factors as an additional explanation for racial inequities (9, 10). Specifically, variation in residential contexts linked to residential segregation shaped by structural racism has been postulated as a contributor to the health gap between Black and White people (9–13).

In Brazil, the spatial concentration of the poor and non-White people is a consequence of urbanization and immigration processes, leading these groups to reside in areas with limited resources (e.g., Brazilian favelas) (7, 14). Residential segregation operates as a fundamental cause of health inequities that generate and reinforce race differences by creating and magnifying differences across neighborhoods in a range of exposures and living conditions that are important to health. Because of structural racism, Brazilian Black and Brown people are more likely than White people to live in racially and economically segregated areas (5, 15), which may contribute to racial inequities in health. It is reasonable, therefore, to hypothesize that more-segregated cities have larger inequities in health by race than less-segregated cities.

Our study addresses an important gap: the limited research on the impact of residential segregation (both racial and economic) on racial inequities in health in Latin America. We used self-rated health (SRH), a subjective measure of health status that has strong predictive value for subsequent morbidity and mortality (16). Using data from a large representative sample from Brazil, we investigated 1) differences in fair or poor SRH by race in 27 Brazilian capital cities, and 2) whether city-level economic or racial residential segregation interacts with race, increasing racial inequities in SRH.

## METHODS

In this cross-sectional study, we used data from the Brazilian National Health Survey (*Pesquisa Nacional de Saúde* (PNS)), a nationwide household-based survey conducted in 2013 by the Ministry of Health and the Brazilian Institute of Geography and Statistics. The aim for the PNS is to describe the health situation and lifestyles of the Brazilian population (17, 18). The 2013 PNS and 2010 Census microdata were obtained from the Brazilian Institute of Geography and Statistics (19, 20).

The PNS sample was representative of Brazil, geopolitical macro-regions, states, metropolitan regions, and the 27 state capitals. Census tracts with at least 60 households were defined as the primary sampling units. Census tracts were selected with probability proportional to the number of households; households were selected by simple random sampling in each primary sampling unit; and 1 adult aged 18 years or older was selected by simple random sampling in each household (21). Of the 93,113 adults sampled, 55,492 were excluded because they were not randomly selected to answer the SRH question (18) and 612 were excluded because they self-declared as being of Asian descent (*n* = 371), as Brazilian indigenous (*n* = 238), or data were missing on their skin color or race (*n* = 3), leaving 37,009 adults in the 27 Brazilian state capitals for analysis. Included and excluded participants were similar, although included participants were slightly older and more likely to be female (Web Table 1) (available at https://doi.org/10.1093/aje/kwac001). The PNS was approved by the National Commission of Ethics in Research.

We defined race as a social construct, a marker of people’s life experiences and social contexts (5, 22–24). Individual-level, self-declared race or skin color was assessed according to the classification officially adopted in the Institute of Geography and Statistics Brazilian Census and most used in Brazilian epidemiologic studies (25): White, Brown (or Pardo, a proxy for persons of mixed White and Black race), Black, Asian (or Yellow, people of Asian descent), or Indigenous (i.e., Brazilian Indigenous) people.

Individual-level SRH was assessed using the following question: “In general, how would you rate your health?” The answers ranged from very good to very poor and were dichotomized as very good/good versus fair/poor/very poor.

Age and gender were self-reported by survey respondents. Individual-level education was also reported by respondents and categorized into university, secondary, primary, and less than primary. Information on self-reported household income per capita was available for 61.9% of the sample (*n* = 22,898) and categorized into tertiles.

City-level residential segregation (economic and racial) was investigated as a modifier of race differences in health. Cities were defined as groups of administrative units that are part of the urban extent as determined from satellite imagery (26). They are akin, therefore, to metropolitan areas. City segregation was assessed using the dissimilarity index, which measures evenness and indicates the percentage of a population group that would have to change residence in order to achieve total integration (27). The index ranges from 0 (complete integration) to 1 (complete segregation) and was calculated for each city according to the following formula:

$$ \begin{equation*}\frac{1}{2}\sum \limits_{i=1}^n\left| \frac{a_i}{A_T}-\frac{b_i}{B_T}\right | \end{equation*}$$

where *n* is the number of census tracts; *A_T_* and *B_T_* are the total populations of the groups being compared at the city area (percentage of households with mean income ≤2 vs. > 2 minimum wages, or percentage of Black and Brown people vs. White people, for income-based or race-based segregation, respectively); and *a_i_* and *b_i_* are their respective populations in census tract *i*. Each census tract (*setor censitário*) contains approximately 250–350 households. The 27 Brazilian cities included 67,588 census tracts, which corresponds to 2,500 census tracts, on average, per city (range, 246–18,955 census tracts). Segregation was categorized into tertiles: low, medium, and high.

Other features of the city social environment were characterized using a city social-environment index (28), constructed by combining *z* scores of city features including the percentages of the following: the population aged 25 years or older who completed primary education or more, households with access to piped water, households with access to a municipal sewage network, and households with more than 3 people per room (inverted).

We compared covariates across categories of SRH and city segregation. Differences were tested using χ^2^ tests and analysis of variance. Multilevel logistic regression models (i.e., individuals nested within cities) with a random intercept for each city and robust variance estimation were used to estimate associations of race with SRH after adjustment. We estimated prevalence ratios (PRs) and prevalence differences (PDs) using the marginal standardization method (based on predicted probabilities of fair/poor SRH from the fitted logistic models) (29–31).

Models were run separately for each dimension of residential segregation: 1) income based and 2) race based. We first estimated associations of race with SRH after adjustment for individual-level age and gender, because these may confound race differences (model 1). For model 2, we added education to model 1 to determine race differences after accounting for differences in individual-level education by race. For model 3, the city-level social-environment index was added to model 2 to account for differences in the social characteristics of cities in which persons of different races live. For models 4A and 4B, we added to model 3 income and racial residential segregation, respectively.

To investigate whether residential segregation modified the association between race and SRH, we added interaction terms between race and segregation (models 5A and 5B). Then, we derived the adjusted marginal prevalence for each race group as well as PRs and PDs for Black and Brown participants, compared with White participants, stratified by levels of segregation. All marginal estimates were standardized to the covariate distribution in the study sample. Because we estimated associations and did not derive prevalence estimates for specific regions, no weights were used in the analyses. In sensitivity analyses, we 1) adjusted data for income in the subsample for which income data were available and 2) stratified data by gender and education.

## RESULTS

The 37,009 adults (41.6% White, 48.2% Brown, 10.2% Black) were distributed in 27 cities with a median of 1,371 respondents per city (range, 641–3,439). The overall prevalence of fair/poor SRH was 31.5%. Black and Brown participants were more likely to report their health as fair/poor than were White participants (Black participants, 36.4%; Brown, 34.0%; White, 27.3%). Those with fair/poor SRH were older and more likely to be female and have less education than those who had good or better health (all *P* < 0.001) (Web Table 2).

The mean dissimilarity indices were 0.32 and 0.25 for income- and race-based segregation, respectively (*r* = –0.08). Greater city residential segregation by income was associated with higher prevalence of fair/poor SRH in survey respondents (Table 1). Cities with more income segregation had larger proportions of Black or Brown respondents and lower proportions of White respondents. The mean age of respondents was also slightly older in cities with greater income segregation, and cities with more income segregation had a higher social environment score.

**Table 1:** Characteristics of the Sample, by Residential Segregation (in Tertiles) (*n* = 37,009), National Health Survey, Brazil, 2013

	**Income Residential Segregation (Index Range)** ^**a**^				**Racial Residential Segregation (Index Range)** ^**b**^			
**Variable**	**Low (0.26–0.30) (*n* = 13,315)**	**Medium (0.31–0.33) (*n* = 11,357)**	**High (0.34–0.41) (*n* = 12,337)**	***P* value**	**Low (0.13–0.21) (*n* = 12,895)**	**Medium (0.22–0.29) (*n* = 12,356)**	**High (0.30–0.34) (*n* = 11,758)**	***P* Value**
Fair/poor self-rated health, %	29.6	30.5	34.4	< 0.001	35.7	29.2	29.2	< 0.001
Race or skin color, %				< 0.001				
White	47.8	41.5	35.2		26.3	41.8	58.2	< 0.001
Brown	45.2	48.3	51.2		65.4	47.6	30.0	
Black	7.0	10.2	13.6		8.3	10.6	11.8	
Age, years^c^	42.1 (16.2)	43.6 (16.6)	43.7 (16.6)	< 0.001	41.3 (16.0)	42.9 (16.3)	45.2 (17.0)	< 0.001
Male gender, %	43.7	40.9	40.7	< 0.001	41.8	42.1	41.6	0.67
Education level, %				0.04				
University	14.8	14.9	15.2		11.0	17.8	16.4	< 0.001
Secondary	37.9	37.0	36.8		38.1	36.9	36.8	
Primary	23.9	23.2	22.9		24.5	22.6	22.9	
Less than primary	23.5	24.8	25.0		26.4	22.7	23.9	
Social environment index^d^	–0.18 (0.63)	0.11 (0.38)	0.12 (0.36)	< 0.001	–0.54 (0.39)	0.19 (0.26)	0.42 (0.10)	< 0.001

^a^ Household income–based dissimilarity index: % of households with mean income ≤2 vs. > 2 minimum wages.

^b^ Race-based dissimilarity index: percentage of Black and Brown people combined vs. percentage of White people.

^c^ Values are expressed as mean (standard deviation).

^d^ Measured at the city level; higher score indicates better social environment.

Respondents living in cities with more racial residential segregation tended to have a lower prevalence of fair/poor SRH (Table 1). They were also more likely to be White and less likely to be Brown than respondents in areas with less racial segregation. The percentage of Black respondents was slightly higher in the highest racial segregation tertile but differences were small. Respondents in more racially segregated cities were also slightly older than those in less-segregated cities, and more racially segregated cities had a higher social environment score (Table 1).

After adjustments for age and gender, the prevalence of fair/poor SRH was 34% higher for Black participants and 25% higher for Brown participants than White participants. Prevalence proportions were 0.09 and 0.07 higher among Black and Brown participants, respectively, compared with White participants (model 1) (Table 2). Additional adjustment for education attenuated PRs and PDs by approximately half, but important differences by race remained (model 2) (Table 2).

**Table 2:** Marginal Prevalence Ratios, Marginal Prevalence Differences, With 95% Confidence Intervals, of Fair/Poor Self-Rated Health Associated With Race and Residential Segregation (in Tertiles) (*n* = 37,009), National Health Survey, Brazil, 2013

**Variable**	**Model 1** ^**a**^		**Model 2** ^**b**^		**Model 3** ^**c**^		**Model 4A** ^**d**^		**Model 4B** ^**e**^	
	**PR**	**95% CI**	**PR**	**95% CI**	**PR**	**95% CI**	**PR**	**95% CI**	**PR**	**95% CI**
Brown (vs. White)	1.25	1.19,1.33	1.12	1.07,1.17	1.12	1.07,1.17	1.11	1.07,1.16	1.12	1.07,1.17
Black (vs. White)	1.34	1.26,1.42	1.18	1.12,1.23	1.18	1.13,1.24	1.18	1.12,1.23	1.18	1.13,1.24
Residential segregation, medium (vs. low)							1.08	0.96,1.22	0.97	0.81,1.16
Residential segregation, high (vs. low)							1.18	1.04,1.32	1.07	0.86,1.33
	**PD**	**95% CI**	**PD**	**95% CI**	**PD**	**95% CI**	**PD**	**95% CI**	**PD**	**95% CI**
Brown (vs. White)	0.07	0.06,0.09	0.03	0.02,0.05	0.03	0.02,0.05	0.03	0.02,0.05	0.03	0.02,0.05
Black (vs. White)	0.09	0.08,0.11	0.05	0.04,0.07	0.05	0.04,0.07	0.05	0.04,0.07	0.05	0.04,0.07
Residential segregation, medium (vs. low)							0.02	–0.01,0.06	–0.01	–0.06,0.04
Residential segregation, high (vs. low)							0.05	0.01,0.09	0.02	–0.05,0.09

Abbreviations: CI, confidence interval; PD, prevalence difference; PR, prevalence ratio.

^a^ Model 1: + age and gender.

^b^ Model 2: + age and gender + education.

^c^ Model 3: + age and gender + education + social environment index.

^d^ Model 4A: + age and gender + education + social environment index + income residential segregation.

^e^ Model 4B: + age and gender + education + social environment index + racial residential segregation.

Additional adjustment for city-level social environment index and income residential segregation (model 4A) did not further attenuate race associations (for Black and Brown participants vs. White participants, respectively: PR = 1.18 (95% confidence interval (CI):1.12, 1.23) and PR = 1.11 (95% CI: 1.07, 1.16); PD = 0.05 (95% CI: 0.04, 0.07) and PD = 0.03 (95% CI: 0.02, 0.05)). Similar findings were observed in models adjusted for racial segregation (model 4B) (for Black and Brown participants vs. White participants, respectively: PR = 1.18 (95% CI: 1.13, 1.24) and PR = 1.12 (95% CI: 1.07, 1.17); PD = 0.05 (95% CI: 0.04, 0.07) and PD = 0.03 (95% CI: 0.02, 0.05)) (Table 2).

In models without interactions between race and segregation, more income residential segregation was associated with higher prevalence of fair/poor SRH (model 4A) (for highest vs. lowest tertile: PR = 1.18 (95% CI: 1.04, 1.32); PD = 0.05 (95% CI: 0.01, 0.09)), whereas racial residential segregation was not (model 4B) (PR = 1.07 (95% CI: 0.86, 1.33); PD = 0.02(95% CI: −0.05, 0.09)) (Table 2).

**Figure 1:**
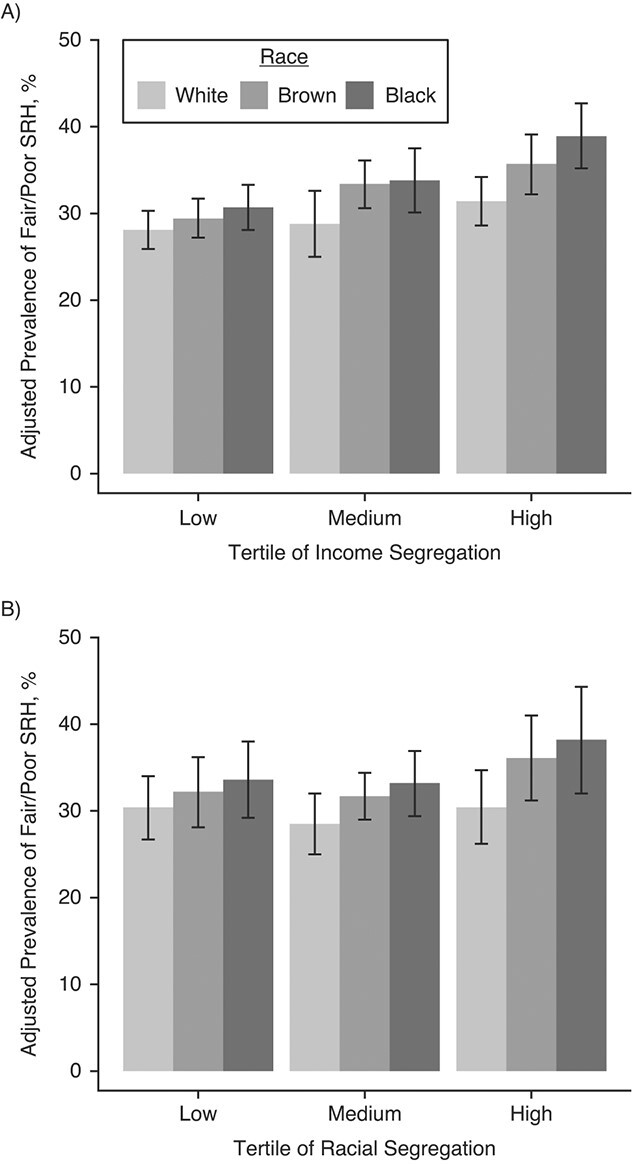
Adjusted marginal prevalence of fair/poor self-rated health by race and residential segregation in tertiles, National Health Survey (*n* = 37,009), Brazil 2013. A) Income segregation, adjusted for age, gender, education, social environment index, income segregation, and interaction term between race and income segregation (*P* for interaction = 0.01). B) Racial segregation, adjusted for age, gender, education, social environment index, racial segregation, and interaction term between race and racial segregation (*P* for interaction = 0.08). Adjusted marginal prevalence data were obtained from fully adjusted models 5A (income segregation) and 5B (racial segregation). SRH, self-rated health.

Multiplicative interactions between race and segregation were statistically significant for income segregation and marginally significant for racial segregation (*P* interaction = 0.01 and 0.08, respectively) (Figure 1A and 1B). Table 3 shows associations of race with SRH stratified by segregation. Overall, race differences in fair/poor SRH were larger in more-segregated than less-segregated cities. For example, the PR for Black versus White participants was 1.09, 1.17, and 1.24, respectively, for cities with low, medium, and high levels of income segregation; and 1.11, 1.16, and 1.25, respectively, cities with for low, medium, and high racial segregation. Similar patterns (with slightly smaller race differences) were observed when comparing Brown participants with White participants (Table 3). The city random-intercept variance was significant and gradually decreased as variables were added but remained significant in full models (data not shown).

**Table 3:** Marginal Prevalence Ratios, Marginal Prevalence Differences, With 95% Confidence Intervals, of Fair/Poor Self-Rated Health Associated With Race, Stratified by Residential Segregation in Tertiles, (*n* = 37,009), National Health Survey, Brazil, 2013^a^

	**Income Segregation** ^**b**^		**Racial Segregation** ^**c**^	
**Variable**	**PR**	**95% CI**	**PR**	**95% CI**
Brown (vs. White)				
At low residential segregation	1.05	0.98, 1.12	1.06	1.00, 1.12
At medium residential segregation	1.16	1.07, 1.26	1.11	1.04, 1.19
At high residential segregation	1.14	1.07, 1.20	1.19	1.12, 1.26
Black (vs. White)				
At low residential segregation	1.09	1.01, 1.19	1.11	1.03, 1.18
At medium residential segregation	1.17	1.10, 1.25	1.16	1.09, 1.25
At high residential segregation	1.24	1.17, 1.31	1.25	1.18, 1.34
	**PD**	**95% CI**	**PD**	**95% CI**
Brown (vs. White)				
At low residential segregation	0.01	–0.01, 0.03	0.02	0.00, 0.04
At medium residential segregation	0.05	0.02, 0.07	0.03	0.01, 0.05
At high residential segregation	0.04	0.02, 0.06	0.06	0.04, 0.08
Black (vs. White)				
At low residential segregation	0.03	0.00, 0.05	0.03	0.01, 0.05
At medium residential segregation	0.05	0.03, 0.07	0.05	0.03, 0.07
At high residential segregation	0.08	0.05, 0.10	0.08	0.05, 0.11

Abbreviations: CI, confidence interval; PD, prevalence difference; PR, prevalence ratio.

^a^ Marginal PRs and PDs were obtained from fully adjusted models 5A (income segregation) and 5B (racial segregation).

^b^ Adjusted for age, gender, education, social environment index, income segregation, and interaction term between race and income segregation.

^c^ Adjusted for age, gender, education, social environment index, racial segregation, and interaction term between race and racial segregation.

In Figure 1A and 1B, we show adjusted marginal prevalence of fair/poor SRH by race for different levels of income and race segregation. Overall, prevalence of fair/poor SRH was higher in Black participants than in White participants, but race differences were greater in more-segregated than in less-segregated cities. There was also a dose–response pattern in prevalence estimates by levels of income segregation for different race groups, such that, in general, the more segregated the city, the greater the adjusted prevalence of fair/poor SRH (Web Table 3). This gradient was present in all 3 race groups for income-based segregation, but the associations were weaker for Whites participants (for cities with the most (vs. least) income segregation, PR = 1.12 (95% CI: 0.98, 1.27), 1.21 (95% CI: 1.07, 1.38), and 1.27 (95% CI: 1.11, 1.45) for White, Brown, and Black participants, respectively) (Web Table 3). More racial segregation was also associated with higher prevalence of fair/poor SRH in Brown and Black participants, but the associations were weaker and not statistically significant (PR = 1.12 (95% CI: 0.90, 1.40) and 1.14 (95% CI: 0.89, 1.45) for the high vs. low segregation tertile in Brown and Black participants, respectively) (Web Table 3).

Additional adjustment for income yielded similar results (Web Table 4). Stratification by gender showed that race differences were larger in women than in men and the magnitude of the effect modification was also stronger in women than in men (Web Table 5). Stratification by education showed that race differences were larger in higher than in lower education strata (Web Table 6). However, a similar pattern of effect modification of race differences by segregation was observed in both genders and in both education strata.

## DISCUSSION

In this large, multiracial population-based sample from Brazil, we found that after adjusting for age, gender, and education, Black people had nearly 20% higher prevalence of fair/poor SRH than did White people, and Brown people had more than 10% higher prevalence of fair/poor SRH than did White people. The excess prevalence of fair/poor SRH was 0.05 and 0.03 among Black and Brown people, respectively, versus White people. These results support our hypothesis of racial inequities in SRH in Brazilian cities. We also found that racial inequities in SRH were larger in more segregated than less segregated areas, for both income and race segregation: the more segregated the cities, the greater the racial disparity in fair/poor SRH.

The link between race and fair/poor SRH can be explained by the effects of structural racism operating over the life course and across generations (23, 32). According to the ecosocial theory of racism and health (32), structural racism creates discriminatory and oppressive social relations, which benefit dominant groups (e.g., White people) and harm subordinated groups (e.g., Black people), thus shaping the distribution of adverse exposures over one’s life course. These exposures become embodied, resulting in the biological expression of racism and, hence, racial health inequities. In Brazil, because of the country’s historical trajectory, a racial hierarchy, imposed since the colonial period by a slave-based economy, fosters racial discrimination and marginalizes the non-White population (5, 33). Still today, long after the end of slavery, racism remains entrenched in Brazilian society.

Evidence of racial inequities in SRH from other Brazilian samples of adults has not always been consistent; in some studies, researchers found racial inequities (22, 34–37), but these were not found in other studies (8, 38). These mixed results might be explained partially by the way in which socioeconomic factors are included in analyses. No race differences in SRH were found in some studies in which SES was adjusted for (8, 38), whereas in other studies in which SES was not adjusted for, race differences were reported (34, 36, 37). In a systematic review on the use of race/ethnicity in epidemiologic studies of race in Brazil, authors reported that in only 27% of the reviewed studies did researchers include SES indicators in their statistical models (25). In our study, we estimated race differences after adjusting for education because education is often posited as a key driver of race differences. However, race differences in education are themselves a manifestation of structural racism; therefore, education can be thought of as a mediator rather than a confounder of race differences. In fact, race differences were about twice as large before education was adjusted for. Education-adjusted results show that in Brazil, race differences persist even after adjusting for education. Additional adjustment for income (in the set of participants for whom income data were available) did not substantially change the results. This finding supports the hypothesis that race affects health through a variety of mechanisms linked to structural racism and its impact on work, neighborhoods, and life-course factors (5, 15, 33, 39). Another important finding from our analysis is that fair/poor SRH was highest among Black and lowest among White participants, with Brown people between those 2 groups, irrespective of residential segregation. Thus, our study findings highlight the importance of analyzing Brown people in a separate category, instead of collapsing them with Black people or excluding them from analysis.

According to Bailey et al. (39), residential segregation is “a foundation of structural racism and contributes to racialized health inequities.” We found that residential segregation interacted with race such that the more segregated the city, the greater the racial gap among Black, Brown, and White people in terms of fair/poor SRH. In other words, living in segregated environments magnifies race differences in health. This is consistent with findings from US studies in which researchers showed that health disparities between Black people and White people were larger in areas with high segregation than in areas with low segregation for several health-related outcomes such as hypertension (40), obesity (41), firearm-associated homicide rates (42), and perinatal outcomes (12, 43). In the Brazilian context, no study has explored racial inequities in health by residential segregation, to our knowledge. Moreover, there appear to be no empirical studies on racial segregation and health and very few on income segregation and health (15, 44). In studies of income segregation and health, researchers have found that more segregation is associated with unhealthy food consumption (44) and with higher prevalence of hypertension and diabetes (15).

A number of processes could explain the link between greater residential segregation and larger race differences. More segregation generates larger differences in the race and income composition of neighborhoods, which, in the context of structural racism and inequality, lead to larger differences across neighborhoods in health-related attributes. As a result, neighborhoods in which the majority of residents are Black or Brown people or have low income are more likely to have health-damaging physical and social environments, including higher levels of toxic exposures, poorer access to health care and other services, greater levels of stressors and violence (11, 39), and unhealthy built environments (e.g., high densities of fast-food restaurants, poor walkability) (45, 46). Residential segregation thus perpetuates structural racism by reinforcing the advantages for White people (5, 45).

Because our hypotheses were about racial health inequities, our main analyses focused on race differences and how they are modified by segregation. Our analyses also allow examination of how segregation is associated with SRH in different racial groups. We found that more income segregation was associated with worse health in all race groups, but stronger associations were observed in Brown and Black people than in White people. Living in more racially segregated cities was also associated with worse health in Black and Brown people (but not in White people), although associations were weaker than those observed for income segregation and were not statistically significant. In comparing the associations of income and race segregation with SRH in this sample, it is important to keep in mind that across the 27 cities, the median percentage of people who were Brown or Black was 60%, whereas the median percentage of people below 2 minimum wages was significantly lower, at 37.4%. Additional work examining various types of segregation and various ways of measuring segregation is needed to better understand how income and race segregation may jointly affect health in the Brazilian context. Comparative work is also needed to explore why racial segregation appeared to be more weakly related to health in our sample than in US samples (12, 40–43).

We also examined whether patterns were different between men and women and generally found larger race differences and stronger effect modification in women than in men. Larger race differences were also found in higher than in lower education strata. The reasons for these differences, as well as other ways in which some of the patterns we observed may be modified by other social and identity characteristics, deserve further exploration.

An important limitation of our study is the cross-sectional design, which limits causal conclusions. We cannot be certain to what extent current levels of segregation reflect exposure to segregation over one’s life, although since relocation in Brazil occurs mostly within the same city (47), cross-sectional residential segregation data might represent past exposure levels. We investigated residential segregation at the city level because of its policy relevance. However, we were unable to directly examine how local segregation (and specific features of segregated areas) affects the health of residents. This may have resulted in underestimates of the effects of segregation on health, because we estimated the overall associations of segregation with health regardless of where an individual actually lived. In future work in Brazil, researchers should consider the analysis of segregation in smaller areas. Strengths of our study include the large survey sample and the inclusion of multiple cities.

In conclusion, we found striking racial inequities in SRH in a large, nationally representative sample of people in Brazil. These differences were reduced but persisted after adjustment for education. In showing evidence of the health gap by skin color or race, our study findings allow us to refute the idea of a Brazilian “racial democracy.” We also demonstrated that these inequities were larger in cities that were more segregated. Racial inequities in health have profound historical roots in Brazil and reflect interpersonal discrimination and structural racism over one’s life course. Our study findings suggest that residential contexts contribute to racial inequities in SRH, because more-segregated cities are likely to have larger differences in the contexts in which persons of different races live. Policies to reduce racial inequities may need to address residential segregation and its consequences for health.

## Supplementary Material

Web_Material_kwac001

## References

[1] Paim J, Travassos C, Almeida C, et al. The Brazilian health system: history, advances, and challenges. *Lancet*. 2011;377(9779):1778–1797. 21561655 10.1016/S0140-6736(11)60054-8

[2] Schmidt MI, Duncan BB, e Silva GA, et al. Chronic non-communicable diseases in Brazil: burden and current challenges. *Lancet*. 2011;377(9781):1949–1961. 21561658 10.1016/S0140-6736(11)60135-9

[3] de Araújo EM, da Costa MCN, Hogan VK, et al. The use of the race/color variable in public health: possibilities and limitations. *Interface - Comun Saúde Educ*. 2009;13(31):383–394.

[4] Freyre G . *Casa-Grande & Senzala: Formacao da Familia Brasileira Sob o Regime da Economia Patriarcal*. Sao Paulo, Brazil: Global Editoria; 2005.

[5] Telles EE . Race in Another America: The Significance of Skin Color in Brazil. Princeton, NJ: Princeton University Press; 2004.

[6] Travassos C, Williams DR. The concept and measurement of race and their relationship to public health: a review focused on Brazil and the United States. *Cad SaúdePública*. 2004;20(3):660–678. 10.1590/s0102-311x2004000300003 15263977

[7] Valente RR, Berry BJL. Residential segregation by skin color: Brazil revisited. *Lat Am Res Rev*. 2020;55(2):207.

[8] Dachs JNW . Factors determining inequalities in the health condition self-assessment in Brazil: analysis of data of PNAD/1998. *Ciênc Saúde Coletiva*. 2002;7(4):641–657.

[9] Subramanian SV, Acevedo-Garcia D, Osypuk TL. Racial residential segregation and geographic heterogeneity in black/white disparity in poor self-rated health in the US: a multilevel statistical analysis. *Soc Sci Med*. 2005;60(8):1667–1679. 15686800 10.1016/j.socscimed.2004.08.040

[10] Finch BK, Do DP, Basurto-Davila R, et al. Does place explain racial health disparities? Quantifying the contribution of residential context to the black/white health gap in the United States. *Soc Sci Med*. 2008;67(8):1258–1268. 18649984 10.1016/j.socscimed.2008.06.018 PMC2614884

[11] Yang T-C, Zhao Y, Song Q. Residential segregation and racial disparities in self-rated health: how do dimensions of residential segregation matter? *Soc Sci Res*. 2017;61:29–42. 27886735 10.1016/j.ssresearch.2016.06.011 PMC5124442

[12] Williams AD, Wallace M, Nobles C, et al. Racial residential segregation and racial disparities in stillbirth in the United States. *Health Place*. 2018;51:208–216. 29715639 10.1016/j.healthplace.2018.04.005 PMC6287738

[13] Mayne SL, Hicken MT, Merkin SS, et al. Neighbourhood racial/ethnic residential segregation and cardiometabolic risk: the multiethnic study of atherosclerosis. *J Epidemiol Community Health*. 2019;73(1):26–33. 30269056 10.1136/jech-2018-211159 PMC6398328

[14] Institute for Applied Economic Research . Residential Segregation and Social Exclusion in Brazilian Housing Markets. Brasília, Brazil: Institute for Applied Economic Research; 2015. (Discussion paper, no. 122). https://hdl.handle.net/10419/220211. Accessed May 17, 2021.

[15] Barber S, Diez Roux AV, Cardoso L, et al. At the intersection of place, race, and health in Brazil: residential segregation and cardio-metabolic risk factors in the Brazilian Longitudinal Study of Adult Health (ELSA-Brasil). *Soc Sci Med*. 2018;199:67–76. 28821371 10.1016/j.socscimed.2017.05.047

[16] Idler EL, Benyamini Y. Self-rated health and mortality: a review of twenty-seven community studies. *J Health Soc Behav*. 1997;38(1):21–37. 9097506

[17] Szwarcwald CL, Malta DC, Pereira CA, et al. National Health Survey in Brazil: design and methodology of application. *Ciênc Saúde Coletiva*. 2014;19(2):333–342. 10.1590/1413-81232014192.14072012 24863810

[18] Damacena GN, Szwarcwald CL, Malta DC, et al. The development of the National Health Survey in Brazil, 2013. *Epidemiol E Serviços Saúde*. 2015;24(2):197–206.

[19] Brazilian Institute of Geography and Statistics . Pesquisa Nacional de Saude 2013. https://www.ibge.gov.br/estatisticas/sociais/saude/9160-pesquisa-nacional-de-saude.html. Accessed on February 12, 2019.

[20] Brazilian Institute of Geography and Statistics . Censo demográfico 2010. https://www.ibge.gov.br/estatisticas/downloads-estatisticas.html. Accessed on July 10, 2020.

[21] de Souza-Júnior PRB, de Freitas MPS, Antonaci GA, et al. Sampling design for the National Health Survey, Brazil 2013. *Epidemiol E Serviços Saúde*. 2015;24(2):207–216.

[22] Chiavegatto Filho ADP, Laurenti R. Racial/ethnic disparities in self-rated health: a multilevel analysis of 2,697 individuals in 145 Brazilian municipalities. *Cad. SaúdePública*. 2013;29(8):1572–1582. 10.1590/0102-311x00139012 24005923

[23] Perreira KM, Telles EE. The color of health: skin color, ethnoracial classification, and discrimination in the health of Latin Americans. *Soc Sci Med*. 2014;116:241–250. 24957692 10.1016/j.socscimed.2014.05.054 PMC4117723

[24] Muniz JO, Bastos JL. Classificatory volatility and (in)consistency of racial inequality. Cad Saúde Pública. 2017;33(suppl 1):e00082816. https://www.scielo.br/scielo.php?script=sci_arttext&pid=S0102-311X2017001305002&lng=pt&tlng=pt. Accessed May 19, 2021. 10.1590/0102-311X00082816 28562697

[25] Kabad JF, Bastos JL, Santos RV. Race, color and ethnicity in epidemiologic studies carried out with Brazilian populations: systematic review on the PubMed database. *Physis Rev Saúde Coletiva*. 2012;22(3):895–918.

[26] Quistberg DA, DiezRoux AV, Bilal U, et al. Building a data platform for cross-country urban health studies: the SALURBAL study. *J Urban Health*. 2019;96(2):311–337. 30465261 10.1007/s11524-018-00326-0 PMC6458229

[27] Massey DS, Denton NA. The dimensions of residential segregation. *Soc Forces*. 1988;67(2):281.

[28] Bilal U, Hessel P, Perez-Ferrer C, et al. Life expectancy and mortality in 363 cities of Latin America. *Nat Med*. 2021;27(3):463–470. 33495602 10.1038/s41591-020-01214-4 PMC7960508

[29] Naimi AI, Whitcomb BW. Estimating risk ratios and risk differences using regression. *Am J Epidemiol*. 2020;189(6):508–510. 32219364 10.1093/aje/kwaa044

[30] Muller CJ, MacLehose RF. Estimating predicted probabilities from logistic regression: different methods correspond to different target populations. *Int J Epidemiol*. 2014;43(3):962–970. 24603316 10.1093/ije/dyu029 PMC4052139

[31] Santos CAS, Fiaccone RL, Oliveira NF, et al. Estimating adjusted prevalence ratio in clustered cross-sectional epidemiological data. *BMC Med Res Methodol*. 2008;8(1):80. 19087281 10.1186/1471-2288-8-80 PMC2625349

[32] Krieger N . Discrimination and health inequities. *Int J Health Serv*. 2014;44(4):643–710. 25626224 10.2190/HS.44.4.b

[33] Hasenbalg CA . *Race Relations in Post-Abolition Brazil: The Smooth Preservation of Racial Inequalities* [dissertation]. Berkeley, CA: University of California; 1978.

[34] Barros MBA, Zanchetta LM, de Moura EC, et al. Self-rated health and associated factors, Brazil, 2006. *Rev Saúde Pública*. 2009;43(suppl 2):27–37. 19936496 10.1590/s0034-89102009000900005

[35] Szwarcwald CL, Damacena GN, de Souza Júnior PRB, et al. Determinants of self-rated health and the influence of healthy behaviors: results from the National Health Survey, 2013. *Rev Bras Epidemiol*. 2015;18(suppl 2):33–44. 27008601 10.1590/1980-5497201500060004

[36] de Sousa JL, Alencar GP, Antunes JLF, et al. Markers of inequality in self-rated health in Brazilian adults according to sex. *Cad Saúde Pública*. 2020;36(5):e00230318. 32490914 10.1590/0102-311x00230318

[37] Kochergin CN, Proietti FA, César CC. Slave-descendent communities in Vitória da Conquista, Bahia State, Brazil: self-rated health and associated factors. *Cad SaúdePública*. 2014;30(7):1487–1501. 10.1590/0102-311x00141213 25166945

[38] Pavão ALB, Werneck GL, Campos MR. Self-rated health and the association with social and demographic factors, health behavior, and morbidity: a national health survey. *Cad Saúde Pública*. 2013;29(4):723–734. 23568302

[39] Bailey ZD, Krieger N, Agénor M, et al. Structural racism and health inequities in the USA: evidence and interventions. *Lancet*. 2017;389(10077):1453–1463. 28402827 10.1016/S0140-6736(17)30569-X

[40] Kershaw KN, Diez Roux AV, Burgard SA, et al. Metropolitan-level racial residential segregation and black-white disparities in hypertension. *Am J Epidemiol*. 2011;174(5):537–545. 21697256 10.1093/aje/kwr116 PMC3202148

[41] Bower KM, Thorpe RJ, Yenokyan G, et al. Racial residential segregation and disparities in obesity among women. *J Urban Health*. 2015;92(5):843–852. 26268731 10.1007/s11524-015-9974-z PMC4608933

[42] Wong B, Bernstein S, Jay J, et al. Differences in racial disparities in firearm homicide across cities: the role of racial residential segregation and gaps in structural disadvantage. *J Natl Med Assoc*. 2020;112(5):518–530. 32641258 10.1016/j.jnma.2020.05.014

[43] Mehra R, Keene DE, Kershaw TS, et al. Racial and ethnic disparities in adverse birth outcomes: differences by racial residential segregation. *SSM Popul Health*. 2019;8:100417. 31193960 10.1016/j.ssmph.2019.100417 PMC6545386

[44] Lopes MS, Caiaffa WT, Andrade ACS, et al. Disparities in food consumption between economically segregated urban neighbourhoods. *Public Health Nutr*. 2020;23(3):525–537. 31839024 10.1017/S1368980019003501 PMC10200452

[45] Williams DR, Collins C. Racial residential segregation: a fundamental cause of racial disparities in health. *Public Health Rep*. 2001;116(5):404–416. 12042604 10.1093/phr/116.5.404 PMC1497358

[46] Diez Roux AV, Mair C. Neighborhoods and health. *Ann N Y Acad Sci*. 2010;1186(1):125–145. 20201871 10.1111/j.1749-6632.2009.05333.x

[47] Friche AAL, Xavier CC, Proietti FA, et al. Saúde urbana em Belo Horizonte. Belo Horizonte, Brazil: Editora da Universidade Federal de Minas Gerais; 2015:160.

