# Supplementary material for: Racial Inequities in Self-Rated Health Across Brazilian Cities: Does Residential Segregation Play a Role?
Source: Am J Epidemiol. 2022 Mar 4;191(6):1071–80. doi: 10.1093/aje/kwac001 (PMC9169054; doi:10.1093/aje/kwac001)
Supplement: Web_Material_kwac001 [file web_material_kwac001.pdf]

Web Material

Title: Racial inequities in self-rated health across Brazilian cities: does residential segregation play a role?

Joanna MN Guimarães, Goro Yamada, Sharrelle Barber, Waleska Teixeira Caiaffa, Amélia Augusta de Lima Friche, Mariana Carvalho de Menezes, Gervasio Santos, Isabel Santos, Leticia de Oliveira Cardoso, Ana V Diez Roux.

Web Tables 1–6

Web Table 1. Comparison Between Included and Excluded, from the Overall PNS Sample (N=93,113), Brazil 2013.

**Variables**	**Included (n=37,009)**	**Excluded (n=56,104)**	***P*-value**
%	39.7	60.3	
Race/skin color, %			
White	41.6	39.3	< 0.001
Brown	48.2	48.8	
Black	10.2	9.4	
Asian[a]	0.0	1.5	
Indigenous[a]	0.0	1.0	
Age in years[b]	43.1 (16.5)	40.5 (15.0)	< 0.001
Gender, male (%)	41.9	49.1	< 0.001
Education, (%)			
University	14.9	14.0	< 0.001
Secondary	37.3	40.3	
Primary	23.4	23.4	
Less than Primary	24.4	22.3	

[a] Not eligible for analysis.

[b] Values are expressed as mean (standard deviation).

Web Table 2. Characteristics of the Sample, Overall and by Self-rated Health Status. National Health Survey (PNS) (N=37,009), Brazil 2013.

		Self-rated health		
**Variables**	**All (n=37,009)**	**Good or Better (n=25,357)**	**Fair/Poor (n=11,652)**	***P*-value**
%	100.0	68.5	31.5	
Race/skin color, %				
White	41.6	44.2	36.2	< 0.001
Brown	48.2	46.4	52.1	
Black	10.2	9.4	11.8	
Age in years[a]	43.1 (16.5)	40.1 (15.3)	49.6 (17.0)	< 0.001
Gender, male %	41.9	44.4	36.4	< 0.001
Education, %				
University	14.9	18.5	7.3	< 0.001
Secondary	37.3	42.1	26.7	
Primary	23.4	22.8	24.6	
Less than Primary	24.4	16.6	41.4	
Social environment index[b]	0.01	0.03 (0.49)	-0.04 (0.50)	< 0.001

[a] Values are expressed as mean (standard deviation).

[b] Measured at the city-level, higher score indicates better social environment.

Web Table 3. Marginal Prevalence Ratios, Marginal Prevalence Differences and 95%CI of Fair/Poor Self-rated Health Associated with Residential Segregation in tertiles, Stratified by Race. National Health Survey (PNS) (N=37,009), Brazil 2013.

**Variables**	**White people**		**Brown people**		**Black people**	
Prevalence Ratios	PR	95%CI	PR	95%CI	PR	95%CI
Income segregation[a]						
Low	1.00	Referent	1.00	Referent	1.00	Referent
Medium	1.03	0.88,1.20	1.13	1.01,1.27	1.10	0.96,1.26
High	1.12	0.98,1.27	1.21	1.07,1.38	1.27	1.11,1.45
Racial segregation[b]						
Low	1.00	Referent	1.00	Referent	1.00	Referent
Medium	0.94	0.77,1.15	0.98	0.83,1.17	0.99	0.81,1.20
High	1.00	0.80,1.26	1.12	0.90,1.40	1.14	0.89,1.45
Prevalence Differences	PD	95%CI	PD	95%CI	PD	95%CI
Income segregation[a]						
Low	0.00	Referent	0.00	Referent	0.00	Referent
Medium	0.01	-0.04,0.05	0.04	0.00,0.07	0.03	-0.01,0.08
High	0.03	-0.00,0.07	0.06	0.02,0.10	0.08	0.04,0.13
Racial segregation[b]						
Low	0.00	Referent	0.00	Referent	0.00	Referent
Medium	-0.02	-0.08,0.04	-0.00	-0.06,0.05	-0.00	-0.07,0.06
High	0.00	-0.07,0.07	0.04	-0.04,0.12	0.05	-0.04,0.13

Abbreviations: PR, Prevalence Ratio; PD, Prevalence Difference; CI, Confidence interval.

Note: Marginal prevalence ratios and prevalence differences obtained from full-adjusted models 5A (Income segregation[a]) and 5B (Race-segregation[b]).

[a] adjusted for age, gender, race, education, social environment index and interaction term between income residential segregation and race.

[b] adjusted for age, gender, race, education, social environment index and interaction term between racial residential segregation and race.

Web Table 4. Marginal Prevalence Ratios, Marginal Prevalence Differences and 95%CI of Fair/Poor Self-rated Health Associated with Race, Stratified by Residential Segregation in tertiles, Additionally Adjusted for Income. National Health Survey (PNS) (N=22,898), Brazil 2013.

	**Full-adjusted + income[c] (N=22,898)**			
	**Income segregation[a]**		**Racial segregation[b]**	
Prevalence Ratios	PR	95%CI	PR	95%CI
Brown (vs White)				
at Low residential segregation	1.04	0.97,1.11	1.05	0.99,1.11
at Medium residential segregation	1.15	1.06,1.25	1.10	1.03,1.18
at High residential segregation	1.13	1.06,1.20	1.18	1.11,1.26
Black (vs White)				
at Low residential segregation	1.08	1.00,1.18	1.10	1.03,1.17
at Medium residential segregation	1.17	1.10,1.25	1.16	1.08,1.24
at High residential segregation	1.23	1.16,1.30	1.25	1.17,1.32
Prevalence Differences	PD	95%CI	PD	95%CI
Brown (vs White)				
at Low residential segregation	0.01	-0.01,0.03	0.02	-0.00,0.03
at Medium residential segregation	0.04	0.02,0.07	0.03	0.01,0.05
at High residential segregation	0.04	0.02,0.06	0.06	0.04,0.08
Black (vs White)				
at Low residential segregation	0.02	0.00,0.05	0.03	0.01,0.05
at Medium residential segregation	0.05	0.03,0.07	0.05	0.02,0.07
at High residential segregation	0.07	0.05,0.09	0.08	0.05,0.10

Abbreviations: PR, Prevalence Ratio; PD, Prevalence Difference; CI, Confidence interval.

Note: Marginal prevalence ratios and prevalence differences obtained from full-adjusted models 5A (Income segregation[a]) and 5B (Racial segregation[b]).

[a] adjusted for age, gender, education, income, social environment index, income segregation and interaction term between race and income segregation.

[b] adjusted for age, gender, education, income, social environment index, racial segregation and interaction term between race and racial segregation.

Web Table 5. Marginal Prevalence Ratios, Marginal Prevalence Differences and 95%CI of Fair/Poor Self-rated Health Associated with Race, Stratified by Residential Segregation in tertiles and Gender. National Health Survey (PNS) (N=37,009), Brazil 2013.

	**Men (N=15,491)**				**Women (N=21,518)**			
	**Income segregation[a]**		**Racial segregation[b]**		**Income segregation[a]**		**Racial segregation[b]**	
Prevalence Ratios	PR	95%CI	PR	95%CI	PR	95%CI	PR	95%CI
Brown (vs White)								
at Low residential segregation	0.98	0.91,1.06	0.99	0.88,1.11	1.09	1.01,1.17	1.10	1.05,1.16
at Medium residential segregation	1.08	0.98,1.19	1.04	0.96,1.13	1.21	1.07,1.37	1.15	1.07,1.25
at High residential segregation	1.05	0.94,1.17	1.08	1.00,1.18	1.19	1.11,1.27	1.25	1.12,1.40
Black (vs White)								
at Low residential segregation	0.98	0.86,1.12	1.04	0.88,1.23	1.16	1.06,1.28	1.15	1.03,1.28
at Medium residential segregation	1.07	0.96,1.20	1.12	0.99,1.28	1.24	1.12,1.37	1.19	1.10,1.29
at High residential segregation	1.26	1.12,1.40	1.21	1.10,1.33	1.23	1.13,1.35	1.29	1.17,1.41
Prevalence Differences	PD	95%CI	PD	95%CI	PD	95%CI	PD	95%CI
Brown (vs White)								
at Low residential segregation	-0.01	-0.02,0.01	-0.00	-0.03,0.03	0.03	0.00,0.05	0.03	0.01,0.05
at Medium residential segregation	0.02	-0.01,0.05	0.01	-0.01,0.03	0.06	0.02,0.10	0.05	0.02,0.07
at High residential segregation	0.01	-0.02,0.04	0.02	-0.00,0.05	0.06	0.04,0.09	0.08	0.04,0.13
Black (vs White)								
at Low residential segregation	-0.01	-0.04,0.03	0.01	-0.04,0.06	0.05	0.02,0.08	0.05	0.01,0.09
at Medium residential segregation	0.02	-0.01,0.05	0.03	-0.00,0.07	0.07	0.04,0.10	0.06	0.03,0.08
at High residential segregation	0.07	0.04,0.11	0.06	0.02,0.09	0.08	0.04,0.11	0.09	0.05,0.13

Abbreviations: PR, Prevalence Ratio; PD, Prevalence Difference; CI, Confidence interval.

Note: Marginal prevalence ratios and prevalence differences obtained from full-adjusted models 5A (Income segregation[a]) and 5B (Racial segregation[b]).

[a] adjusted for age, education, social environment index, income segregation and interaction term between race and income segregation.

[b] adjusted for age, education, social environment index, racial segregation and interaction term between race and racial segregation.

Web Table 6. Marginal Prevalence Ratios, Marginal Prevalence Differences and 95%CI of Fair/Poor Self-rated Health Associated with Race, Stratified by Residential Segregation in tertiles and Education. National Health Survey (PNS) (N=37,009), Brazil 2013.

	**<= Primary education (N=17,673)**				**>= Secondary education (N=19,336)**			
	**Income segregation[a]**		**Racial segregation[b]**		**Income segregation[a]**		**Racial segregation[b]**	
Prevalence Ratios	PR	95%CI	PR	95%CI	PR	95%CI	PR	95%CI
Brown (vs White)								
at Low residential segregation	1.03	0.96,1.11	1.06	1.00,1.12	1.14	1.00,1.29	1.15	1.05,1.27
at Medium residential segregation	1.10	1.00,1.20	1.03	0.97,1.09	1.39	1.20,1.61	1.36	1.19,1.55
at High residential segregation	1.09	1.02,1.16	1.15	1.09,1.21	1.31	1.21,1.41	1.39	1.23,1.57
Black (vs White)								
at Low residential segregation	1.06	0.96,1.17	1.11	1.03,1.20	1.24	0.95,1.63	1.19	0.96,1.48
at Medium residential segregation	1.10	1.00,1.22	0.98	0.93,1.03	1.41	1.10,1.81	1.73	1.40,2.13
at High residential segregation	1.12	1.02,1.23	1.20	1.14,1.27	1.62	1.48,1.79	1.50	1.33,1.68
Prevalence Differences	PD	95%CI	PD	95%CI	PD	95%CI	PD	95%CI
Brown (vs White)								
at Low residential segregation	0.01	-0.02,0.04	0.02	-0.00,0.05	0.02	0.00,0.04	0.03	0.01,0.05
at Medium residential segregation	0.04	0.00,0.08	0.01	-0.01,0.04	0.07	0.04,0.09	0.06	0.04,0.07
at High residential segregation	0.04	0.01,0.07	0.06	0.04,0.09	0.06	0.04,0.08	0.07	0.04,0.11
Black (vs White)								
at Low residential segregation	0.02	-0.02,0.06	0.05	0.01,0.08	0.04	-0.01,0.09	0.04	-0.01,0.09
at Medium residential segregation	0.04	-0.00,0.09	-0.01	-0.03,0.01	0.07	0.02,0.12	0.11	0.06,0.16
at High residential segregation	0.05	0.01,0.10	0.09	0.06,0.12	0.12	0.09,0.15	0.09	0.05,0.13

Abbreviations: PR, Prevalence Ratio; PD, Prevalence Difference; CI, Confidence interval.

Note: Marginal prevalence ratios and prevalence differences obtained from full-adjusted models 5A (Income segregation[a]) and 5B (Racial segregation[b]).

[a] adjusted for age, gender, social environment index, income segregation and interaction term between race and income segregation.

[b] adjusted for age, gender, social environment index, racial segregation and interaction term between race and racial segregation.
